# SARS‐CoV‐2 modulation of RIG‐I‐MAVS signaling: Potential mechanisms of impairment on host antiviral immunity and therapeutic approaches

**DOI:** 10.1002/mef2.29

**Published:** 2022-12-11

**Authors:** Mingming Wang, Yue Zhao, Juan Liu, Ting Li

**Affiliations:** ^1^ State Key Laboratory of Quality Research in Chinese Medicines, Macau Institute for Applied Research in Medicine and Health Macau University of Science and Technology Macau China; ^2^ Guangdong Provincial Key Laboratory of Medical Molecular Diagnostics, Department of Clinical Immunology, Institute of Clinical Laboratory Medicine Guangdong Medical University Dongguan China

**Keywords:** antiviral immunity, RIG‐I‐MAVS signaling, SARS‐CoV‐2, therapeutic approaches

## Abstract

The coronavirus disease 2019 (COVID‐19) is a global infectious disease aroused by RNA virus severe acute respiratory syndrome coronavirus 2 (SARS‐CoV‐2). Patients may suffer from severe respiratory failure or even die, posing a huge challenge to global public health. Retinoic acid‐inducible gene I (RIG‐I) is one of the major pattern recognition receptors, function to recognize RNA viruses and mediate the innate immune response. RIG‐1 and melanoma differentiation‐associated gene 5 contain an N‐terminal caspase recruitment domain that is activated upon detection of viral RNA in the cytoplasm of virus‐infected cells. Activated RIG‐I and mitochondrial antiviral signaling (MAVS) protein trigger a series of corresponding immune responses such as the production of type I interferon against viral infection. In this review, we are summarizing the role of the structural, nonstructural, and accessory proteins from SARS‐CoV‐2 on the RIG‐I‐MAVS pathway, and exploring the potential mechanism how SARS‐CoV‐2 could evade the host antiviral response. We then proposed that modulation of the RIG‐I‐MAVS signaling pathway might be a novel and effective therapeutic strategy to against COVID‐19 as well as the constantly mutating coronavirus.

## Graphical abstract

The retinoic acid‐inducible gene I mitochondrial antiviral signaling (RIG‐I‐MAVS) axis is designated as a major signaling pathway in the innate immune response for RNA viruses. This review summarized the role of various proteins derived from severe acute respiratory syndrome coronavirus 2 (SARS‐CoV‐2) on the RIG‐I‐MAVS pathway and explored the underlying mechanism of SARS‐CoV‐2 to escape the host antiviral response. Regulation of RIG‐I‐MAVS pathway might be a potentially therapeutic strategy to boost immunity against COVID‐19.

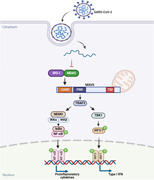

## 1 INTRODUCTION

Coronavirus disease 2019 (COVID‐19) mediated by severe acute respiratory syndrome coronavirus 2 (SARS‐CoV‐2) has swept the globe, threatening human health and safety.^1^, ^2^ SARS‐CoV‐2, SARS‐CoV in 2002, and Middle East respiratory syndrome coronavirus (MERS‐CoV) in 2012 are the three lethal respiratory human coronavirus as well as the highest pathogenic human respiratory coronaviruses (CoVs).^3^, ^4^, ^5^ According to the reports of WHO, the mortality rate of SARS‐COV and MERS‐CoV is 15% and 35%, respectively.^6^ COVID‐19 exhibits high infectivity, which not only has shaken health care but also sharply affected the economic structure all of the world.

The main feature of COVID‐19 is upper and lower respiratory tract infection, including asymptomatic patients and mild, moderate, severe, and critical patients in the infected population. Mild symptoms mainly manifested as fever and cough, while moderate symptoms progressed to pneumonia and local inflammation. In addition, symptoms of severe or critical infection can progress to pneumonia, disseminated intravascular coagulation, acute respiratory distress syndrome, and ultimately lead to multiorgan infection dysfunction and mortality. Notably, SARS‐CoV‐2 is capable of transmission from asymptomatic or presymptomatic individuals with high infectious efficiency, and significant viral shedding was observed during incubation, mainly owing to the specific CoV pathogenesis and host antiviral immunity.^7^, ^8^ Therefore, the antiviral immunity of the infected host is closely correlated to the prognosis of the disease.

Immunity is a physiological protective function of the body, innate immunity is the host first line of defense against pathogens. It mainly plays a crucial role in resistance to viral infection. Innate immunity can initiate pathogen‐associated molecular patterns (PAMPs) that are often unique molecular features not found in host cells, it primarily through its associated receptors specific recognition of single‐stranded RNA, double‐stranded RNA (dsRNA), DNA or as well as viral genomes, resulting in a signaling cascade. As a pattern recognition receptor of the innate immune protection system, retinoic acid‐inducible gene I (RIG‐I) owns great worth in the control of RNA virus infection, mainly recognize dsRNA, which activates intracellular immune signaling pathways resulting in the antiviral response.^9^, ^10^, ^11^

By analyzing RIG‐I‐like receptors (RLRs) in the control of RNA virus infection, we provided a hint of the immune response mediated by SARS‐CoV‐2 and furnished a therapeutic strategy to deal with COVID‐19 as well as RNA virus‐mediated diseases.

## 2 THE STRUCTURE OF SARS‐COV‐2

Viruses have a complex correlation with their hosts and employ various strategies to evade host cells. Immune evasion is one of the unique features of SARS‐CoV‐2 attributed to its unique protein structure. As a member of the CoVs family, SARS‐CoV‐2 is a characteristic single‐stranded positive‐sense RNA virus. CoVs are divided into four genera assigned as α, β, γ, and δ. CoVs of genus β are further separated into four subgroups of A, B, C, and D. As current report, SARS‐CoV and SARS‐CoV‐2 belong to the β genus B subgroup, and MERS‐CoV belongs to the β genus Subgroup C. SARS‐CoV‐2 shares 79.5% similarity with SARS‐CoV and 50% homology with MERS‐CoV in genetic sequence.^12^ The genome size of SARS‐CoV‐2 is approximately 29.7 kb. SARS‐CoV‐2 is a globular of 80–120 nm in size wrapped in a phospholipid bilayer with nucleocapsid (N) protein and a genomic RNA core, characterized by a spike (S) protein projected on the outer surface. The viral genome of SARS‐CoV‐2 has 14 open reading frames (ORFs), which are divided into ORF1a and ORF1b, mainly located at the 5′ end, accounting for two thirds of the full length. These genomes can produce 16 nonstructural proteins (Nsp1–Nsp16), participating in the replication and transcription of the virus in the host, controlling enzymatic activities, and assembling the replication transcription complex (RTC). During infection, 16 nonstructural proteins are presented in ORF1a and attached to RTC, whereas distributed in the cytosol are proteins expressed by ORF1b.^13^ Coupling of capping machinery in SARS‐CoV‐2 RTC is critical for viral mRNA maturation and gene transcription. Nsp1–Nsp16 are essential for viral replication and have some immune evasion functions.

In addition, the 3′ end exists structural protein and several accessory proteins, which are necessary for genome expression, accounting for one‐third of the full length. Among them, structural proteins are composed of spike (S), membrane (M), envelope (E), and nucleocapsid (N). The S protein is a heavily glycosylated protein, that can be embedded in the viral envelope, which mainly mediates the entry of the virus. The Furin protease cleavage site (PRRAR) cleaves the S protein into two subunits, the N‐terminal S1 subunit, and the membrane‐bound C‐terminal S2 subunit. The S1 subunit consists of the well‐recognized receptor binding domain (RBD) of angiotensin‐converting enzyme 2, and the S2 subunit is responsible for promoting fusion between the virus and the host cell. RBD is the most variable structure in SARS‐CoV‐2.^14^ M and E proteins are responsible for assembling the virus and participating in the formation of the mature virus envelope. SARS‐CoV‐2 assembly occurs in the lumen of the endoplasmic reticulum (ER) Golgi intermediate compartment.^15^ N protein protects the viral RNA genome at the core of the virus and has the capability to combine with the RNA virus and package it into new virus particles.

**Figure 1:**
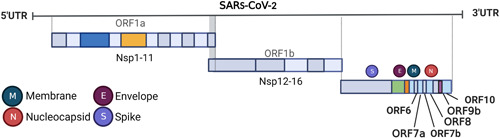
The structure of SARS‐CoV‐2. SARS‐CoV‐2 genome are are composed of 4 structural proteins (spike, membrane, envelope, and nucleocapsid), 14 open reading frames (ORFs), and 16 nonstructural proteins (Nsp1–Nsp16).

SARA‐CoV‐2 contains many genes concentrated in the 3′ region of the genome that encodes accessory proteins, which encompass ORF3a, ORF3b, ORF6, ORF7a, ORF7b, ORF8, ORF9b, ORF9c, and ORF10 (Figure 1).^8^, ^9^, ^10^ Although these accessory proteins are not essential for viral replication, they are thought to play a role in regulating host‐infected cellular metabolism and antiviral immunity.^16^, ^17^, ^18^ Besides involving in viral invasion, replication, and proliferation, SARS‐CoV‐2 has evolved multiple mechanisms to weaken and evade the host innate immune response during the evolutionary process of rivalry with the host.

## 3 RIG‐I‐MITOCHONDRIAL ANTIVIRAL‐SIGNALING PROTEIN (MAVS) SIGNALING

RLRs are pattern‐recognition receptors,^19^ which are mainly distributed in innate immune cells, such as macrophages, neutrophils, and dendritic cells. As an RNA helicase located in the cytoplasm with a highly helical structure, RLRs are used to recognize RNA viruses. The RLRs family consists of RIG‐I, melanoma differentiation‐associated protein 5 (MDA5), and laboratory of genetics and physiology 2 (LGP2).^20^, ^21^ They are sharing the central DECH‐box helicase domain and a C‐terminal regulatory domain (CTD). Among them, the DECH‐box domain contains the conserved helicase subdomains Hel1 and Hel2, which are essential for ATP hydrolysis and subsequent RNA recognition as well as signal transduction.^22^, ^23^

The crystal structure reveals the interaction between CTD of the RLRs and the 5′‐terminal region of RNA.^24^, ^25^ RIG‐I preferentially recognizes 5′ triphosphate or diphosphate double‐stranded RNA (dsRNA) and prefers relatively short dsRNAs (less than 1 kbp), while MDA5 binds longer dsRNAs (greater than 1 kbp) and forms nucleoprotein filaments.^26^, ^27^ It was further reported that only the RIG‐I CTD specifically recognized and bind to 5′ terminal SARS‐CoV‐2.^28^, ^29^ Distinguished from LGP2, RIG‐I and MDA5 have two caspase activation and recruitment domains (CARDs) at the N‐terminus, and each of them have approximately 85 amino acids. This region interacts with the CARD domain of MAVS to facilitate downstream signaling.

**Figure 2:**
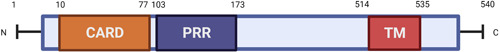
The structure of mitochondrial antiviral‐signaling (MAVS). MAVS contains three domains: an N‐terminal caspase activation and recruitment domain (CARD), a middle proline‐rich region (PRR), and a C‐terminal transmembrane (TM) domain.

The mitochondrial membrane‐associated protein MAVS serves as a core adaptor protein for RLR signaling and is essential for controlling viral infection. MAVS is a 540‐amino acid mitochondrion‐resident protein encoded by the nuclear genome containing an N‐terminal CARD, proline‐rich region (PRR), and C‐terminal transmembrane (TM) domain (Figure 2).^30^ The TM region of MAVS is anchored to the outer membrane of mitochondria and peroxisomes, while the CARDs are located in the cytoplasm and can interact with RIG‐I/MDA5.^31^, ^32^ Normally, the two CARD domains of RIG‐I are masked by intramolecular interactions with the helicase domain, which, upon binding to viral RNA, is exposed to interact with the single CARD domain of MAVS.^24^, ^31^, ^33^, ^34^

**Figure 3:**
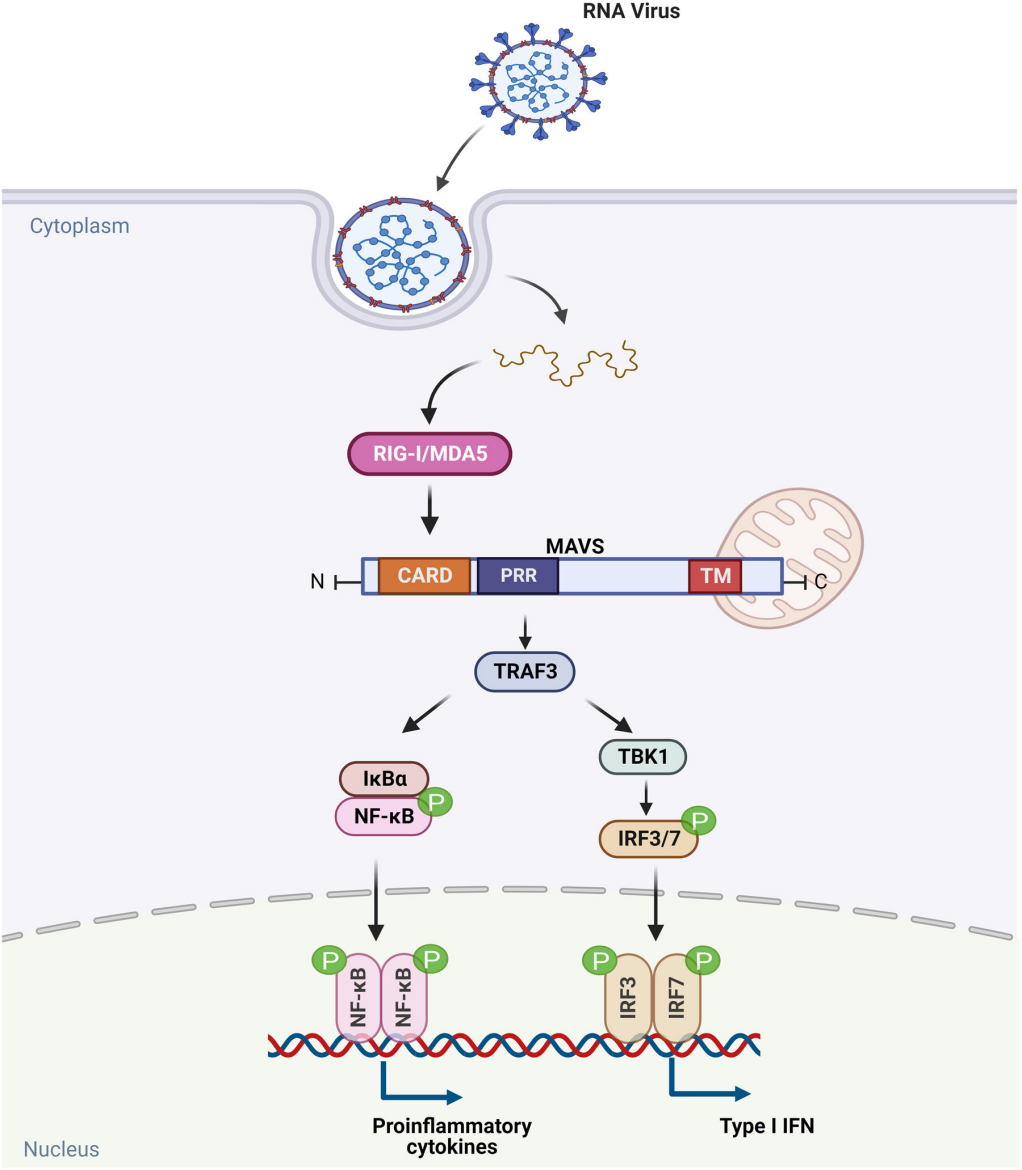
RLR signaling pathways. Binding of viral components to RIG‐I and MDA5 induces their interaction with MAVS via their common CARDs. MAVS activates NF‐κB pathway and IRF3 and IRF7 pathway induces the expression of type I IFN genes. CARD, caspase activation and recruitment domain; IFN, interferon; IRF, interferon regulatory factor; MAVS, mitochondrial antiviral signaling; MDA5, melanoma differentiation‐associated protein 5; RIG‐I, retinoic acid‐inducible gene I; RLR, RIG‐I‐like receptor.

The aggregation or oligomerization of CARDs plays an important role in the RIG‐I/MDA5 and MAVS signaling pathways. Induction of CARD oligomerization can be mediated by K63‐linked polyubiquitin chains (K63‐Ubn), or by filament formation of RNA‐binding domains (helicase domain and CTD).^35^, ^36^ Among them, RIG‐I is covalently modified with K63‐Ubn by an E3 ligase, Trim25.^36^, ^37^ Compared to RIG‐I, the mechanism by which MDA5 oligomerizes its 2CARD and stimulates MAVS filament formation is still unclear. MAVS forms highly ordered filamentous aggregating on the mitochondrial surface after RIG‐I/MDA5 activation via CARD‐CARD domain resulting in the recruitment of downstream signaling. The aggregated adaptor MAVS in turn triggers two signaling cascades such as tumor necrosis factor receptor (TNFR)‐associated factor 3 (TRAF3) and inhibitor of NF‐κB kinase, leading to the activation of interferon regulatory factor 3 (IRF3), IRF7, NF‐κB as well as type I interferon (IFN‐I) signaling (Figure 3). Eventually, the production of pro‐inflammatory cytokine and immune response are induced. As IFN is essential for antiviral immunity, these findings indicated that suppression of RIG‐I‐MAVS signaling contributes to the RNA virus infection. Accordingly, existing studies have confirmed that IFN are significantly impaired in severe COVID‐19 patients.^8^, ^32^, ^38^

## 4 THE ROLE OF STRUCTURAL PROTEINS OF SARS‐COV‐2 IN RIG‐I‐MAVS PATHWAY

### 4.1 M protein of SARS‐CoV‐2 and RIG‐I‐MAVS pathway

M protein is composed of 222 amino acids, consisting of three domains N‐terminus, triple‐TM, and C‐terminal cytoplasmic. M protein is essential for the assembly and release of viral particles and is the most abundant envelope protein to wrap RNA genome of SARS‐CoV‐2 by interacting with nucleocapsid (N) protein. M protein primarily localized to the mitochondrial, ER, and Golgi, which are the significant platform in innate antiviral immunity.

**Figure 4:**
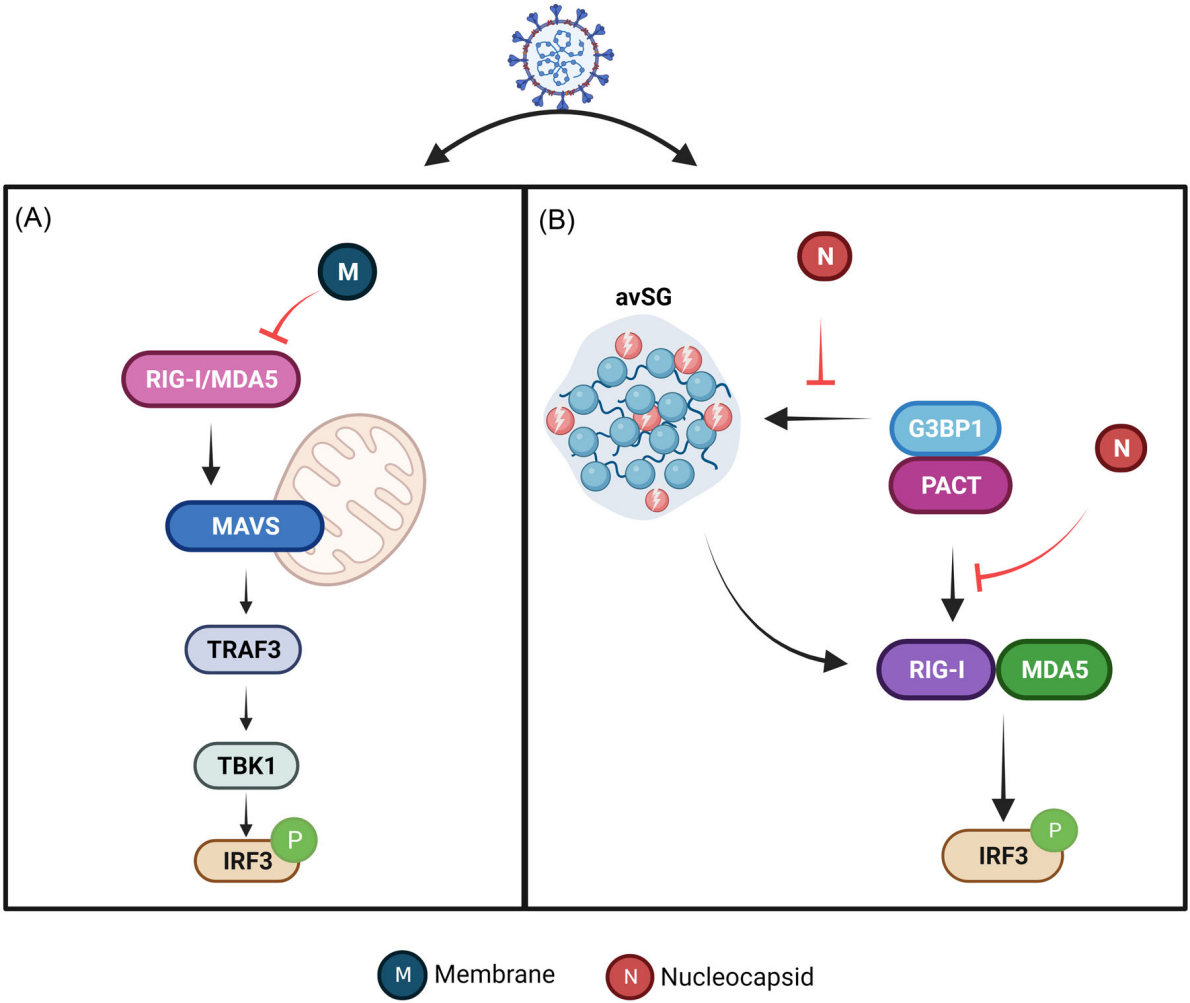
The function of SARS‐CoV‐2 structural proteins on RIG‐I‐MAVS signaling pathway. (A) SARS‐CoV‐2 M impedes the formation of RIG‐I ‐MAVS signaling pathway. (B) SARS‐CoV‐2 N protein inhibits RIG‐I‐MAVS signaling pathway by targeting G3BP1 and PACT. MAVS, mitochondrial antiviral signaling; RIG‐I, retinoic acid‐inducible gene I.

As one of the structural protein of SARS‐CoV‐2, M protein inhibits the production of IFN mediated by RIG‐I and MDA5‐MAVS signaling via sensing the cytosolic dsRNA.^35^ By interacting with the central adaptor protein MAVS, M protein could impair MAVS aggregation and then affect the downstream TRAF3 and TANK binding kinase1 (TNF‐α activated protein kinase 1 or TBK1)‐IRF3 signaling pathway, leading to attenuation of the innate antiviral response (Figure 4A).^36^ These studies revealed that interfering with the interaction between M protein and MAVS is a potential therapeutic strategy to treat SARS‐CoV‐2 infection. In addition, TBK1 is a key kinase in RIG‐I signaling, activated by an RNA sensor, and its activity must be tightly controlled to maintain proper IFN production. Ubiquitination of TBK1 is a crucial mechanism to regulate its activity. It was revealed that M protein interacts with TBK1 and promotes TBK1 degradation through K48‐linked ubiquitination, eventually inhibits IFN production to generate antiviral responses.^37^

### 4.2 N protein of SARS‐CoV‐2 and RIG‐I‐MAVS pathway

Nucleocapsid protein N of SARS‐CoV‐2 is encoded in the 9th ORF of the virus and consisted of 419 amino acids to form a helical nucleocapsid by packaging the newly produced RNA genome. In addition, N protein localizes to RTC early in infection, which is a multifunctional RNA‐binding protein.^39^ N protein consists of two RNA‐binding domains, N‐terminal domain (NTD) and CTD.^38^ The fundamental function of the protein lies in participation in the replication and assembly of SARS‐CoV‐2 and disturbs the production of IFN of the host.^40^

N proteins interact with RNA and other key host cell proteins by forming biomolecular condensates such as stress granules (SGs). As antiviral hubs, SGs involved in the shutdown of protein synthesis and the recruitment of innate immune signaling intermediates.^41^ They are found in the cytoplasmic aggregates of mRNA and RNA binding protein, which is responsible for translation inhibition and limitation of viral infection. SGs have been validated as the critical terrace for triggering RIG‐I and MDA5 signaling pathways.

GTPase‐activating protein SH3‐domain‐binding protein 1 (G3BP1), an SG core protein is an essential antiviral protein involved in the innate immune response.^42^ As the nucleating protein of SGs, G3BP1 acts as a positive regulator of the RIG‐I‐ signaling pathway to mediate antiviral responses. However, N protein quarantines G3BP1 from forming anti‐viral stress granule, which further prevents the cofactors G3BP1 and PACT to trigger RIG‐I. Additionally, the N protein impaired the recognition of dsRNA by RIG‐I and promoted viral replication (Figure 4B).^43^ Hence, suppression of N protein expression probably contributes to the activation of MAVS signaling, and then produces antiviral effect.

## 5 THE ROLE OF NONSTRUCTURAL PROTEINS OF SARS‐COV‐2 IN RIG‐I‐MAVS PATHWAY

### 5.1 Nsp1 protein of SARS‐CoV‐2 and RIG‐I‐MAVS pathway

Nsp plays a pivotal role in the life cycle of SARS‐CoV‐2 by constituting the RTC of the virus. Nsp1 is a 180 amino acid nonstructural protein composed of an NTD, a C‐terminal helix, and a short linker region.^44^ Because Nsp1 is encoded by the gene closest to the 5′ end of the viral genome, it is rapidly expressed after the virus enters the cell. Meanwhile, as a multifunctional and leader protein, Nsp1 can participate in inhibiting the expression of host proteins.^45^, ^46^ Nsp1 directly positions the C‐terminal helical domain in the mRNA entry channel of the 40S ribosomal subunit, preventing transcript entry into the ribosome and shutting down host protein synthesis.^47^, ^48^ Studies have shown that translation shutdown of Nsp1 almost completely suppresses the innate immune response. The eIF3 complex is central to the eukaryotic translation initiation process. As its subunit, eIF3j can bind to the 40S ribosomal subunit. Experiments have shown that Nsp1 competes with eIF3j for binding to the 40S ribosomal subunit and impairs the ability of eIF3 to bind to the 40S subunit,^49^, ^50^ which blocks the eIF3 complex in translation initiation, implying that Nsp1 contributes to cellular antiviral translational shutdown.^47^, ^51^, ^52^, ^53^

More importantly, Nsp1 uses multiple strategies to interfere with IFN production and its downstream signaling. Nsp1 can not only block MAVS‐induced IFN production,^23^, ^54^ but also partially prevent IFN induction by blocking IRF3 phosphorylation. As a critical part of innate immunity, IFN plays a key role in the early response to virus infection, especially respiratory virus infection. If the type I IFN response is delayed, it will lead to an increase in viral load. Inhibition of Nsp1 produces IFN secretion is effective against viral infections.^55^

### 5.2 Nsp5 protein of SARS‐CoV‐2 and RIG‐I‐MAVS pathway

**Figure 5:**
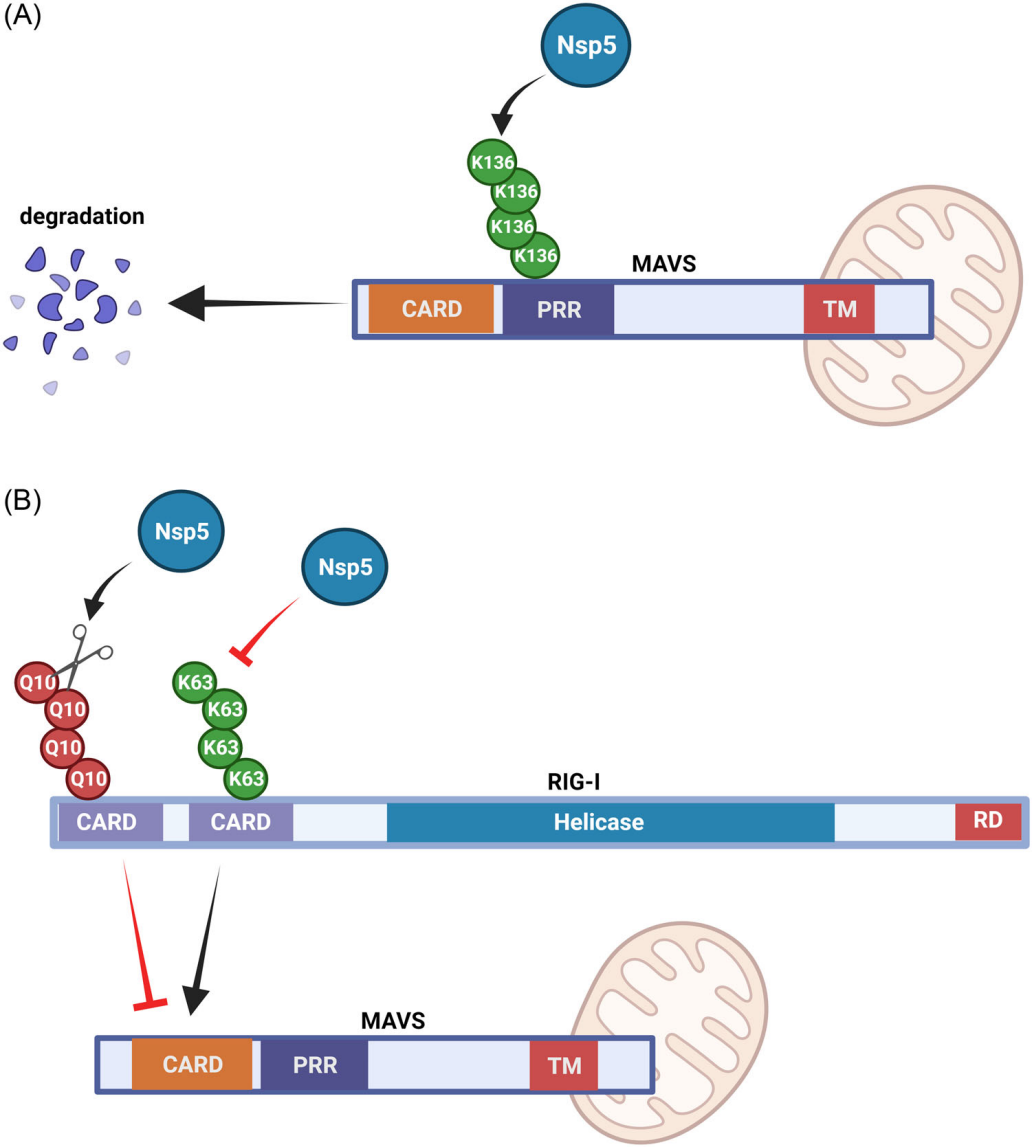
The function of SARS‐CoV‐2 Nsp5 protein on RIG‐I‐MAVS signaling pathway. (A) SARS‐CoV‐2 Nsp5 cleaves RIG‐I at the Q10 residue or promotes RIG‐I degradation interference RIG‐I signaling pathway. (B) SARS‐CoV‐2 Nsp5 targets the K136 residue of MAVS to promote protein degradation. MAVS, mitochondrial antiviral signaling; RIG‐I, retinoic acid‐inducible gene I.

Nsp5, known as the major protease or 3C‐like protease, recognizes more than 11 cleavage sites to produce Nsp4‐Nsp16. Nsp5 directly mediates the maturation of Nsps and determines the viral life cycle in host cells. As a cysteine protease, Nsp5 can process most SARS‐CoV‐2 nonstructural proteins to produce functional components and provide a viable target for antiviral inhibition. In addition, Nsp5 cleaves the most N‐terminal 10 amino acids (Q10 residues) in RIG‐I and deprive its ability to activate MAVS. (Figure 5A). As an E3 ligase, Nsp5 targets the K136 residue and induce ubiquitin‐proteosome‐mediated degradation of MAVS to restrict IFN expression by decreasing K63‐linked ubiquitination on RIG‐I (Figure 5B).^56^, ^57^, ^58^

Because it is highly conserved among CoVs, Nsp5 not only is recommended as a potential target for drug discovery against SARS‐CoV‐2 but also served as a potential strategy for discovering the drugs with a broad‐spectrum antiviral.^59^, ^60^ Two electrophilic warhead‐containing small‐molecule inhibitors, 2CN113 and 2CN115 were synthesized.^58^ The two compounds not only covalently bind to the catalytic residues of cysteine proteases and attenuate Nsp5‐mediated RIG‐I and MAVS disruption to restore activation of the innate immune response, but also reduce SARS‐CoV‐2 nonstructural protein processing, ultimately preventing SARS‐CoV‐2 replication.

### 5.3 Nsp6 and Nsp12 protein of SARS‐CoV‐2 and RIG‐I‐MAVS pathway

Nsp6 is a membrane protein of approximately 34 kDa, with eight TM helices and highly conserved C‐terminus. Nsp6 could facilitate viral replication by rearranging the cell membrane to form double‐membrane vesicles,^61^ and antagonize the innate immune response by binding to TBK1 to inhibit IRF3 phosphorylation.^57^, ^62^

Nsp12 is another Nsp of SARS‐CoV‐2, which is composed of 932 amino acids with two conserved domains, the NiRAN and the polymerase domains. Nsp12 exhibits poor processivity in RNA synthesis and is required to form a complex with Nsp7 and Nsp8 for RNA‐dependent RNA polymerase (RdRP) activity to confer the processivity of Nsp12. Different from Nsp6, Nsp12 inhibits IRF3 nuclear translocation rather than phosphorylation to attenuate IFN production as well antiviral response. Interestingly, Nsp6 blocking MAVS‐induced IFN production is independent of Nsp12 polymerase activity.^63^

### 5.4 Nsp13, Nsp14, Nsp15, and Nsp16 protein of SARS‐CoV‐2 and RIG‐I‐MAVS pathway

Nsp13, Nsp14, and Nsp15 of SARS‐CoV‐2 are associated with vesicle trafficking pathways. Nsp13, a key enzyme in viral replication is highly conserved among all CoVs. Nsp13 composed of 601 amino acids possesses RNA 5′‐triphosphatase activity and is responsible for introducing the 5′‐end cap of viral mRNA. The 5′‐end cap is the translation recognition site, which plays a key role in viral proliferation. Nsp13 has helicase activity, which is required for the unwinding of dsRNA substrates. The helicase activity of Nsp13 could be enhanced via binding to Nsp12. Notably, Nsp13 inhibits IFN production by recruiting TBK1 to p62 for autophagic degradation, enabling it to avoid clearance by the host innate immune response.^64^, ^65^

Unlike Nsp13, Nsp14 is a conserved 60 kDa bifunctional enzyme consisting of 527 amino acids that retain 3′–5′ exonuclease (ExoN) and N7‐MTase activities. The ExoN domain is located at N‐terminal and provides proofreading activity to remove mismatched nucleotides introduced by viral RdRPs.^66^, ^67^ Due to the large genome of CoVs, proofreading activity of the ExoN domain is critical for maintaining the integrity of the viral RNA.^68^ Substantial evidence supports that ExoN can block the activation of dsRNA sensors such as IFN‐β and TNF‐αexpression.^69^ In addition, the S‐adenosylmethionine (SAM)‐dependent N7‐MTase domain is located at the C‐terminal and plays a key role in viral RNA 5′ capping.^70^, ^71^ The 5′ cap protects the stability and translation of viral mRNA, allowing the virus to evade the host's innate immune response.^72^, ^73^ Meanwhile, Nsp10 interacts with the Nsp14 ExoN domain and enhances its activity.^67^, ^74^ Both enzymatic domains are essential for successful viral replication, which makes Nsp14 an attractive drug target.^75^, ^76^ Nsp14 may mediate escape from immune surveillance by activating the IFN pathway and activate pro‐inflammatory responses by altering the expression of NF‐κB and CXCL8.^77^, ^78^ As a virally encoded translational repressor, Nsp14 shuts down the synthesis of antiviral proteins of the host. Global translational repression by Nsp14 results in the elimination of IFN‐I‐dependent induction of IFN‐stimulated genes.^79^ In summary, Nsp14 deserves further investigation as a potent IFN antagonist.^77^, ^80^

Besides Nsp14, Nsp15 is also considered to be an IFN antagonist.^81^ Nsp15 consists of 347 amino acids, is a 3′–5′ exoribonuclease, and provides an extra fidelity to the RTC complex, by way of proofreading function in RNA replication. Ring finger protein 41 (RNF41)/neuregulin receptor degradation protein 1 (Nrdp1) belong to E3 ubiquitin ligase, which is targeted by Nsp15 protein. Nsp15 could repress IFN expression by interacting with TBK1 and IRF3 activator RNF41 and Nrdp1.^82^, ^83^ Furthermore, Nsp15 contains a uridylate‐specific endoribonuclease (NendoU) structure,^84^ which is highly conserved among CoVs.^85^ It contains C‐terminal catalytic domain and functions as RNA endonucleases on phosphodiesters and hydroxyl termini. The NendoU domain promotes the catalytic activity of Nsp15, indicating that the domain could be applied as a potentiate antiviral drug target to treat COVID‐19.^86^

Although Nsp15 is not required for viral RNA synthesis, it is a key component of CoVs pathogenesis.^87^ It was reported that Nsp15 colocalizes with membrane‐associated viral replication complexes.^88^ and the viral dsRNA in membrane‐associated replication complex can protect it from detection by host sensors,^87^ implying that Nps15 could mediate viral evasion from host dsRNA sensors (RLRs). Additionally, mutations cause Nsp15 to become destabilized or even abolish endonuclease activity, stimulate MDA5‐dependent IFN production and activate host dsRNA sensors, however, the mechanism by which Nsp15 affects dsRNA sensor activation remains unknown. One hypothesis is that, similar to pestiviruses and Lassa viruses, SARs‐CoV‐2 degrades viral dsRNA by encoding viral ribonucleases. The other hypothesis is that Nsp15 recognizes and cleaves specific dsRNA targets.^89^, ^90^ The cleavage of Nsp15 can be influenced by RNA structure.^91^ However, the current conclusions were attributed to in vitro studies,^87^ and it is necessary to evaluate Nsp15‐mediated dsRNA cleavage in the context of viral infection in vivo.

Nsp16 is a 2′O‐methyltransferase (2′O‐MTase) that is part of the replication‐transcription complex.^92^ It mimics the human protein Cap‐specific mRNA (nucleoside‐2′‐O‐)‐methyltransferase (CMTr1),^93^ providing a cap structure at the 5′ end of viral mRNAs to increase translation efficiency and prevent them from being captured by the host sensor RIG‐I^94^ or MDA5.^95^ Unlike other 2′O‐MTases that have independent activity, Nsp10 can activate Nsp16 for substrate binding by opening its SAM and RNA‐binding loops.^96^, ^97^, ^98^, ^99^

## 6 THE ROLE OF ACCESSORY PROTEINS OF SARS‐COV‐2 OF IN RIG‐I‐MAVS PATHWAY

ORF6 is a small protein consisting of 61 amino acids and its molecular weight is approximately 7 kDa. The SARS‐CoV‐2 ORF6 gene shares 69% homology with SARS‐CoV ORF6, with the difference being two amino acid deletions at its C‐terminus.^100^ Remarkably, ORF6 protein of SARS‐CoV‐2 demonstrated more efficient innate immune antagonists than that of SARS‐CoV. In line with the difference of ORF6 from SARS‐CoV and SARS‐CoV‐2, the antagonisms of ORF6 rely on its C‐terminal region by inhibiting IRF3 nuclear translocation.^101^ Mechanistic study showed that ORF6 could bind to importin Karyopherin α 2 via its C‐terminus, and then inhibited IRF3 nuclear translocation.^53^, ^62^ Therefore, it could antagonize the host innate immune response by inhibiting RIG‐I signaling pathways and IFN‐I activation.

ORF7a is a TM protein of 121 amino acid residues^102^ that leads to NF‐κB activation through association with TAK1. Recent studies have shown that ORF7a blocks STAT2 phosphorylation, thereby inhibiting IFN‐I signaling.^62^ RNF121 acts as an E3 ligase that induces polyubiquitination of ORF7a.^103^ Ubiquitination by covalently bound ORF7a overwhelms the host ubiquitin system to promote antagonistic IFN‐I responses.^104^

ORF8 gene consists of 121 amino acids^105^ and is part of a hypervariable genomic region which thought to be highly sensitive to nucleotide deletions and substitutions.^106^ It was noted that hallmarks of COVID‐19, such as high levels of systemically released pro‐inflammatory cytokines, chemokines, and growth factors associated with lung injury, were relatively low in Δ382 ORF8 compared with WT‐infected patients. On the contrary, IFN gamma and other cytokines responsible for T cell activation were upregulated.^107^

ORF8, ORF7a, and ORF9 or ORF10 of SARS‐CoV‐2 or SARS‐CoV share the IgSF structure. However, SARS‐CoV‐2 ORF8 differs from its homologs by the lack of a C‐terminal TM domain and the presence of a long insertion within the core.^108^ Studies have found that ORF8 can inhibit the expression of IFN‐β and the activation of IFN‐stimulated response element promoters, which can trigger the RIG‐I/MDA5 pathway.^53^, ^109^ Deletion of the SARS‐CoV‐2 ORF8 gene causes a milder COVID‐19 phenotype, indicating that ORF8 has potential as an antiviral target for SARS‐CoV‐2. However, the hypervariable characteristics and rapid evolution of the ORF8 gene probably impose certain limitations as an antiviral target.^110^ Nevertheless, More than 300 SARS‐CoV‐2‐human protein–protein interactions have been identified, and 47 human proteins have been found to interact with SARS‐CoV‐2 ORF8, of which more than 10 have been clinically tested.^83^

ORF9b is a specific accessory protein of SARS‐CoV‐2 rather than other CoVs. ORF9b has been reported to accumulate in large amounts within 24 h of COVID‐19 patients. Importantly, the antibody responses to ORF9b were found in patients with the viral infection, suggesting the critical role for ORF9b of SARS‐CoV‐2 in host–virus interactions.^111^ TOM70 is responsible for activating antiviral innate immune responses and initiating IFN production. ORF9b interacts with TOM70, which significantly reduces IFN activation.^112^ It was reported that TRAF‐mediated polyubiquitination recruits NEMO to the mitochondrial MAVS signaling, and activates NF‐κB during antiviral signaling.^113^ Upon viral stimulation, as the unique accessory protein of SARS‐CoV‐2, ORF9b could specifically target NEMO and disrupt its K63‐linked polyubiquitin, thereby inhibiting the production of IFN.^4^, ^114^ Hence, ORF9b probably is employed as an antigen to develop a vaccine for the treatment of COVID‐19.

Substantial evidence indicated that the autophagy is not only a process of degradation of harmful components and intermodulates but also participates in MAVS‐mediated antiviral signaling,^115^ suggesting that autophagy is an important host defense mechanism against viral infection. The studies have shown that ORF10 can colocalize with LC3B in mitochondria, further inducing mitophagy and decreased mitochondrial MAVS expression.^42^ The autophagy receptor NIX was also shown to be a specific regulator of ORF10‐induced mitophagy. In detail, NIX is localized on the OMM and directly interacts with LC3 through its LIR motif to mediate mitochondrial clearance.^116^ Therefore, by interacting with NIX and LC3B, ORF10 leads to mitophagy and inhibits the production of IFN‐I as well as other pro‐inflammatory cytokines, which in turn promotes viral replication.^117^, ^118^ Hence, ORF10 involves in the different stages of SARS‐CoV‐2 infection by induction of autophagy.

## 7 CONCLUDING REMARKS

Virus–host interactions underlie the pathogenesis of CoVs and influence innate and adaptive immunity. Innate immunity includes many antimicrobial factors such as IFN‐I and is triggered very early after infection.^119^ Adaptive immunity, consisting of pathogen‐specific antibodies and T cells, develops later and helps clear infection and immunity to subsequent infections. Innate and adaptive immunity work together to detect and eliminate pathogens.

According to statistics, a total of 12,449,443,718 vaccine doses have been administered.^120^ The number of newly confirmed cases of COVID‐19 in the world is still on the rise because its variants continue to appear and the escape of SARS‐CoV‐2 against the host immune defense system. SARS‐CoV‐2 variants enhance viral transmissibility, invisibility, and the risk of reinfection. To keep abreast of the development and changes of SARS‐CoV‐2 mutant strains, it is necessary to maintain sufficiently detailed and systematic monitoring for SARS‐CoV‐2‐genome mutations so that appropriate public health control measures can be taken in a timely manner.

During the early stage of SARS‐CoV‐2 infection, the host immune system is suppressed. Notably, the host innate defense system, including the IFN system, cannot exert its antiviral function in time, providing an opportunity for the early replication of the virus. As an important part of the body immune defense, innate immune system offers a general and crucial protective response against viral invasion by preventing viral replication, promoting viral clearance, repairing tissue injury, as well as stimulating an adaptive immune response.

There are large individual differences in the severity of COVID‐19 patients, including asymptomatic and mildly infected patients, and critically ill patients who face death at any time.^121^ Therefore, individualized medicine is required for these patients. Accumulated evidence has shown that IFN plays a key role in reducing viral proliferation and regulating the host's immune response to viral infection.^122^ In the early stages of SARS‐CoV‐2 infection, if IFN‐I response is robust, the viral load could be fastly controlled, resulting in mild disease Accordingly, if IFN‐I responses are delayed or diminished early in SARS‐CoV‐2 infection, viral replication and spread could be induced, eventually, leading to immunopathological responses, T‐cell lymphopenia, or induction of strong antibody responses. Although the production of autoantibodies to IFN‐I is rare in healthy controls (less than 0.3% frequency) as well as asymptomatic infected individuals, it is observed in at least 10% of critically ill COVID‐19 patients. Besides IFNβ, IFNα intervention may normalize the dysregulated innate immunity of COVID‐19.^123^ In individuals with genetically or serologically deficient IFN‐I, replication of SARS‐CoV‐2 occurs unhindered, resulting in severe and even life‐threatening COVID‐19.^48^

IFN levels in patients are considered to be an important factor affecting disease severity in COVID‐19 patients, and the RIG‐I‐MAVS pathway is the main driver of IFN production.^124^, ^125^ In the review, we not only unravel how RIG‐I is activated and regulated by SARS‐CoV‐2 and gain insights into the pathogenesis of the virus, but also greatly advance our understanding of the viral escaping from the innate immune system. In light of these studies, we found RIG‐I is important for vaccine or drug development against or suppression of COVID‐19 infection. Additionally, RIG‐I‐MAVS signaling is critical in the host's defense against pathogen invasion by mediating antiviral immune response.^30^, ^126^, ^127^, ^128^

**Figure 6:**
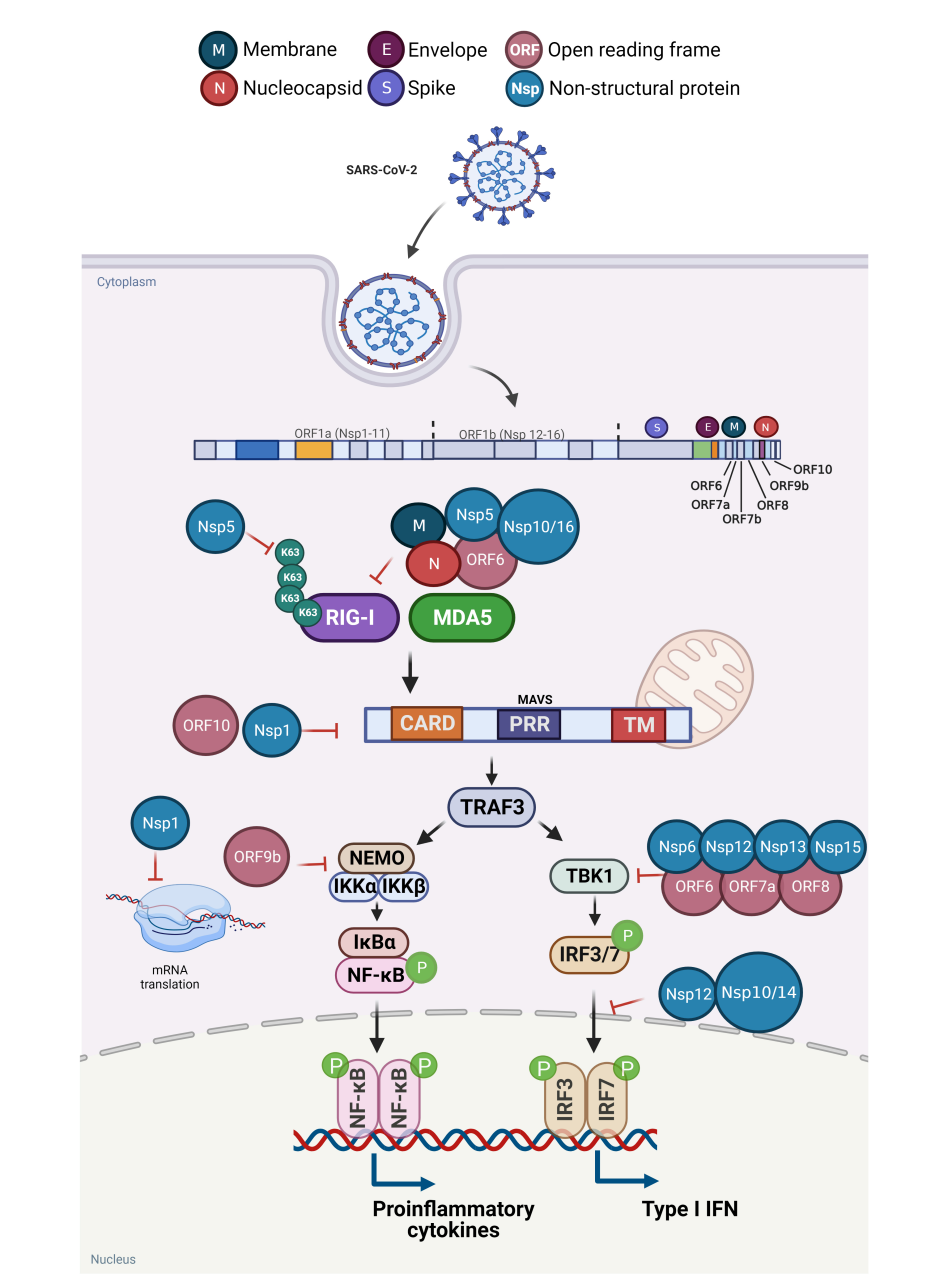
The interaction of SARS‐CoV‐2 proteins on RIG‐I‐MAVS pathway. SARS‐CoV‐2 RNA activates the RIG‐I‐MAVS‐dependent IFN signaling pathway. During SARS‐CoV‐2 infection, various proteins of SARS‐CoV‐2 can act differently on RIG‐I‐MAVS to antagonize the antiviral type I IFN response. IFN, interferon; MAVS, mitochondrial antiviral signaling; RIG‐I, retinoic acid‐inducible gene I.

Host IFN‐I plays a crucial role in limiting SARS‐CoV‐2 infection and replication, while SARS‐CoV‐2 may encode multiple viral proteins to counteract IFN responses, several SARS‐CoV‐2 proteins have been identified as IFN antagonists (Figure 6).^129^ By acting on various molecules in the RIG‐I‐MAVS signaling pathway, SARS‐CoV‐2 viral proteins can evade host antiviral responses and promote viral replication. Since the Spike of SARS‐CoV‐2 antagonizes RIG‐I signaling, interacts with IRF3 and further blocks IFN‐I activation and induction of downstream signaling, it may serve as a key target for therapeutic intervention in COVID‐19.^130^ As an immune evasion factor for SARS‐CoV‐2, Nsp1 effectively interferes with cellular translation machinery, and expanded the scope of viral infection.^131^ As well known, mRNA capping and proofreading play critical roles in SARS‐CoV‐2 replication and transcription.^70^ The mRNA of SARS‐CoV‐2 can be capped and modified under the synergistic action of various nonstructural proteins.^132^ The modified viral mRNA is highly similar to the host mRNA, which reduces the possibility of viral mRNA being recognized by the host immune system. For instance, Nsp16 forms an obligatory complex with Nsp10 that together methylates the 5′ end of viral‐encoding mRNA to mimic cellular mRNA, thereby protecting the virus from host innate immune constraints.^93^, ^132^

In conclusion, we believe that intervention in the interaction between RIG‐I‐MAVS signaling and SARS‐CoV‐2 viral proteins or activation of RIG‐I‐MAVS signaling is a potential therapeutic strategy against viral infection and replication. Studying antiviral strategies from the perspective of innate immunity can greatly expand the understanding of SARS‐CoV‐2 pathogenesis, clarify the escape mechanism of SARS‐CoV‐2 to the host immune system, which will be helpful to design the secondary immune response vaccines and screen the drugs for against COVID‐19.

## AUTHOR CONTRIBUTIONS

Mingming Wang and Ting Li conceived the idea; Mingming Wang and Yue Zhao performed the literature search and drafted the manuscript; Ting Li revised the manuscript; Juan Liu reviewed and edited the manuscript. All authors have read and approved the article.

## CONFLICT OF INTEREST

The authors declare no conflict of interest.

## ETHICS STATEMENT

Not applicable.

## Data Availability

Not applicable.
